# Pancreatic Involvement by Mixed Serous Neuroendocrine Neoplasm Detected by Endosonography With Fine-Needle Tissue Acquisition in Von Hippel-Lindau Disease

**DOI:** 10.7759/cureus.89564

**Published:** 2025-08-07

**Authors:** Anna Carolina Orsini-Arman, Rodolfo Carvalho Santana, Paulo César Galvão do Amaral, Rafael Kemp, José Sebastião Dos Santos, Jose C Ardengh

**Affiliations:** 1 Medical Oncology, Pontificia Universidade Católica de Campinas, Campinas, BRA; 2 Surgical Gastroenterology, Aliança Hospital/Rede D'Or Hospital Group, Salvador, BRA; 3 Surgery and Anatomy, Hospital das Clinicas da Faculdade de Medicina de Ribeirão Preto - Universidade de São Paulo, Ribeirão Preto, BRA; 4 Diagnostic Imaging, Universidade Federal de Sao Paulo, São Paulo, BRA; 5 Digestive Endoscopy Service, Hospital Moriah, São Paulo, BRA

**Keywords:** endosonography, fine needle biopsy, pancreatic neoplasms, pancreatic neuroendocrine tumor, serous cystadenoma, von hippel-lindau disease

## Abstract

The Von Hippel-Lindau disease (VHL) is an autosomal dominant condition characterized by multiple cystic tumors in several organs, including the pancreas. The symptoms are variable, and suspicion must be raised with typical lesions, such as a hemangioblastoma of the central nervous system (CNS) or retina, associated with a renal cell carcinoma, a pheochromocytoma or multiple pancreatic cysts, besides neuroendocrine tumors (NET). The diagnosis in a patient without a family history should be suspected in case of a hemangioblastoma of the CNS and/or retina, which could also be associated with other lesions, such as pancreatic cysts and NETs.

The mixed serous-neuroendocrine neoplasia (MSNN) is a combination of serous cyst neoplasia (SCN) and pancreatic-neuroendocrine tumors (p-NET). There are only a few reports in the literature of this type of neoplasia and the majority are associated with VHL. Based on the distribution pattern of SCN and of p-NETs, it is classified into four subtypes: diffuse, mixed, solitary and coalition. This is an unprecedented report of two cases of VHL, evaluated by endoscopic ultrasound-guided fine needle biopsy (EUS-FNB), which allowed the identification and diagnosis and determined the follow-up of these patients using microhistology (McH) and allowed the differentiation of MSNN, mixed tumors and solitary tumors.

## Introduction

Von Hippel-Lindau disease (VHL) is hereditary. It occurs secondary to germline mutations in the VHL suppressor gene located on the third chromosome, specifically on the p25-26 arm [1]. It is characterized by an autosomal dominant predisposition to develop hemangioblastomas of the retina and central nervous system (CNS), renal cell carcinoma, pheochromocytoma and other tumors with notable phenotypic variability, such as the mixed serous-neuroendocrine neoplasm of the pancreas (MSNN) [1,2]. The VHL gene is manifested by a variety of benign and malignant tumors, which may initially present in childhood, adolescence or adulthood, manifesting lesions in the CNS, vasculature, retina and pancreas. The germline mutations in the VHL gene are heterogeneous and widely distributed throughout the coding sequence, being detectable in all families with VHL [3].

To date, numerous pancreatic lesions have been reported in patients with VHL, including serous cyst neoplasia (SCN) [4], pancreatic-neuroendocrine tumor (p-NET) [5,6], adenocarcinomas [7], hemangioblastomas [1], metastasis of renal cell carcinoma [8] and MSNN [9]. The MSNN is a combination of SCN and p-NETs [10]. There are only a few reports in the medical literature of this combination worldwide and most of them are associated with VHL [11]. The MSNN was first described in the 2010 World Health Organization (WHO), being classified as part of the pancreatic tumors. Regarding its pathogenesis, the literature agrees with the combined origin of cells derived from endocrine and exocrine differentiation [9]. Genetically, its development suggests the possibility of a common genetic basis with VHL. The MSNN is particularly suspicious in the presence of multiple SCNs in the pancreas. Based on the distribution pattern of SCNs and p-NET, the MSNN is classified into four subtypes: (1) Diffuse: the entire pancreas is occupied by SCNs associated with one or more p-NETs, located in any region of the pancreas; (2) Mixed: two different tumors combine together into a single mass and cannot be divided distinctly; (3) Solitary: two different components are present in the pancreas without any mixing; and (4) Coalition: two types of neoplasms separate from each other in most of the pancreatic area, but with a partially mixed or overlapping zone [2].

VHL typically involves the pancreas, often with clinical follow-up for asymptomatic patients, if they are identified and differ histologically from other types of cystic tumors, which may be malignant and/or have malignant potential [12]. Pancreatic involvement varies between 17% and 56% [1,13,14]. Previously, it was thought that pancreatic lesions were not clinically relevant because they tended to be asymptomatic when compared to other lesions found in VHL [3]. However, large cysts compress neighboring organs [4] and p-NETs can metastasize [5]. Isolated pancreatic lesions can confirm the diagnosis of VHL in family members of patients affected by this disease, even in the absence of detectable mutations [3]. Generally, only one type of pancreatic lesion is present and, even with multiple cystic lesions, it is rarely associated with endocrine or exocrine pancreatic insufficiency [1]. The coexistence of two synchronous pancreatic lesions is rare and studies show that p-NETs have a lower malignant potential in patients with VHL than in the general population, in addition to a lower potential for metastases [1]. Nevertheless, the risk of malignancy still requires periodic surveillance.

The authors report two patients with VHL with multiple benign cysts, identified by imaging and endoscopic ultrasound-guided fine needle biopsy (EUS-FNB). The fragments of tissue were sent for microhistology (McH) technique. In both cases, SCNs of different sizes were identified, scattered throughout the pancreatic gland, associated with p-NETs, one mixed tumor and one solitary tumor.

## Case presentation

Clinical case 1

**Figure 1:**
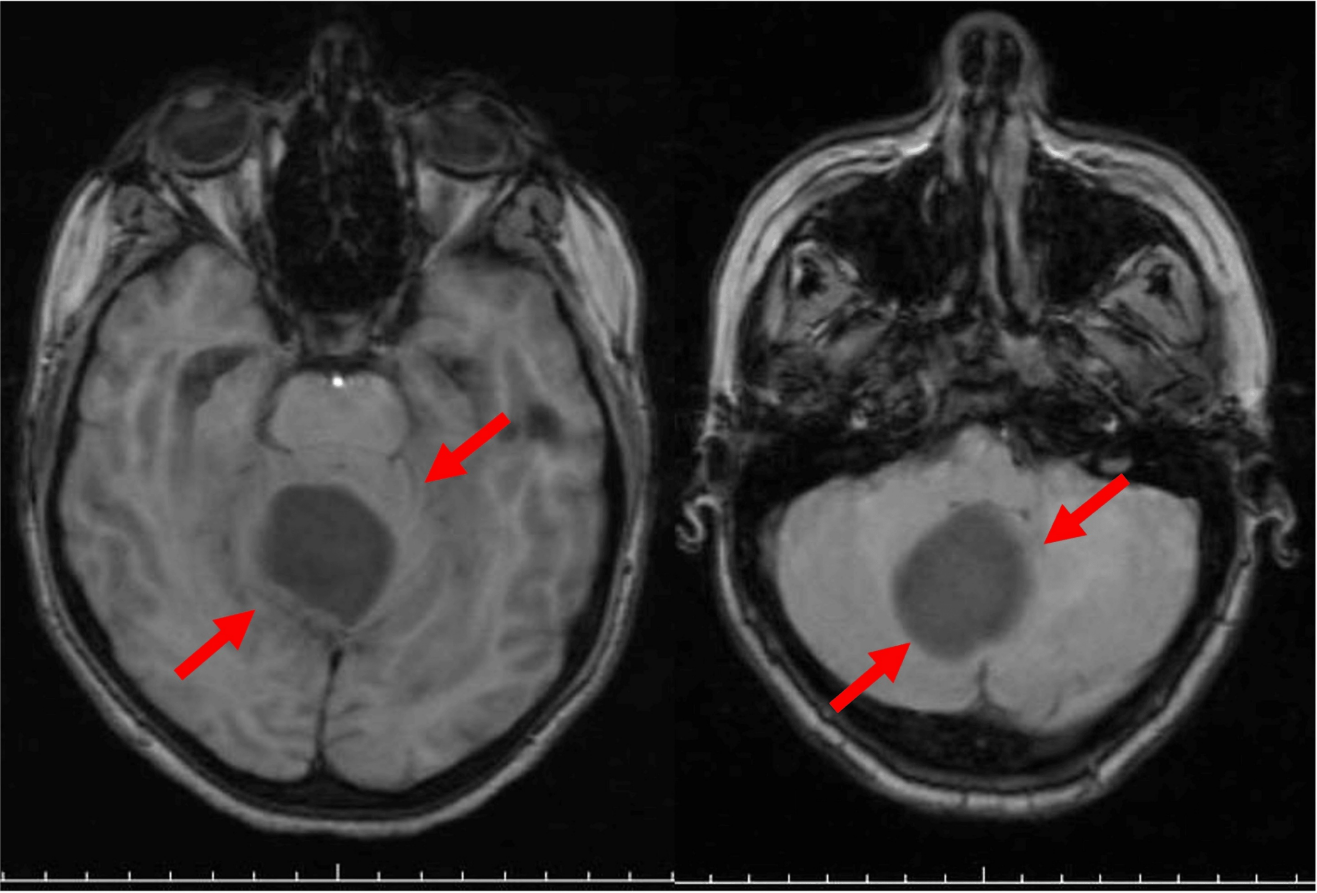
Brain MRI: note the hypointense nodule centered in the IV ventricle, shaping the cerebellar parenchyma (red arrows).

A 23-year-old woman presented with a holocranial headache associated with intermittent vomiting for two months. She developed dizziness, imbalance, and blurred vision one month before diagnosis. A few days before the consult, she had been experiencing postprandial fullness and epigastric pain. The patient is a smoker, social drinker and has bilateral polycystic ovarian disease. The neurological examination revealed balance changes, and the abdominal examination showed mild pain in the right hypochondrium on palpation. Laboratory tests were normal. The brain magnetic resonance imaging (MRI) showed an expansive lesion in the posterior fossa of the cerebellum with mild signs of intracranial hypertension (Figure 1).

**Figure 2:**
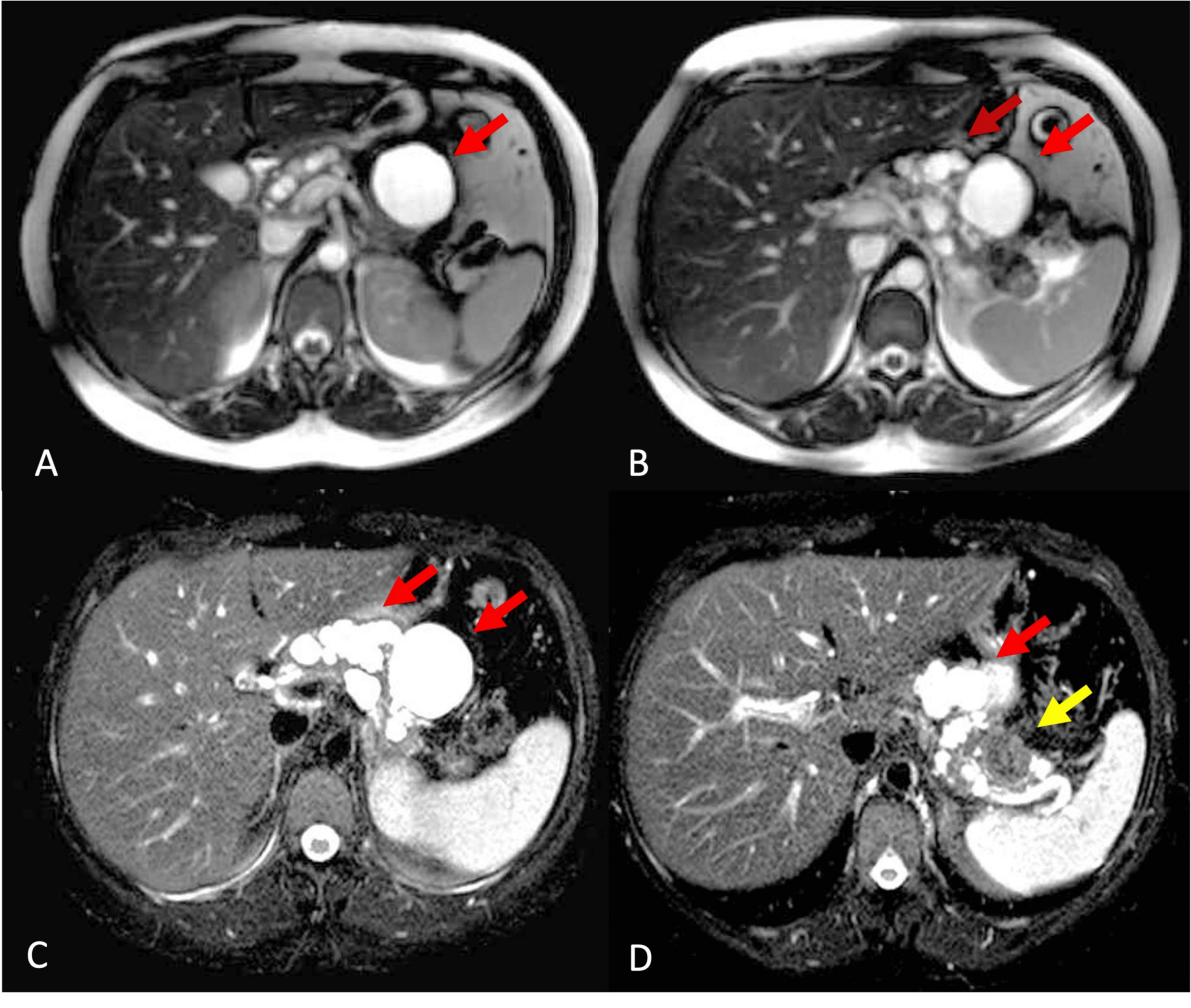
MRI without T2-weighted fat-saturation: (A and B) multiple hyperintense areas, with different forms scattered throughout the pancreatic gland (red arrows). With fat-weighted saturation (C and D): note the multiple cystic areas scattered throughout the pancreatic parenchyma (C). (D) Hypointense, rounded, heterogeneous nodule, with tiny hyperintense areas in between (yellow arrow) located in the tail of the pancreas.

**Figure 3:**
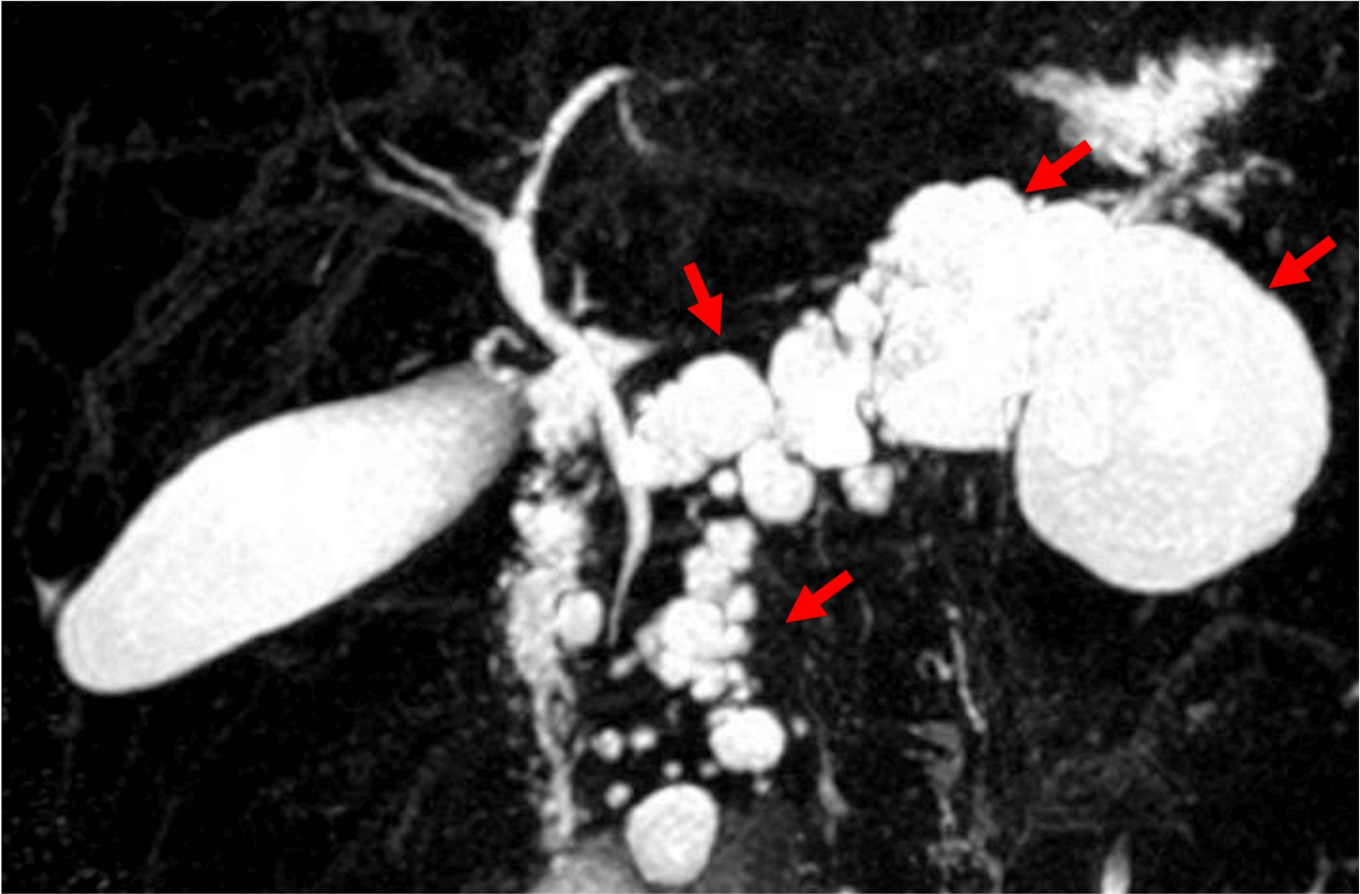
Magnetic resonance cholangiopancreatography (MRCP): pancreatic gland with numerous cystic formations of different sizes occupying practically the entire parenchyma (red arrows). Unable to visualize the main pancreatic duct.

The patient was referred for ophthalmological evaluation and the fundus examination showed hemangioma in the retina, confirmed by ultrasound of the orbit. Once VHL was confirmed, craniotomy with excision of the cerebellar lesion was performed and the diagnosis of hemangioblastoma was confirmed by pathology. The patient presented asymptomatic after neurosurgical treatment and was referred to the pancreas group at the Hospital (Ribeirão Preto Medical School) for follow-up. The abdominal MRI and the magnetic resonance cholangiopancreatography (MRI/MRCP) showed a preserved, although shapeless, pancreatic parenchyma. Also, it displayed cystic formations with lobulated contours, some with thin septations, replacing practically the entire parenchyma, in addition to a nodular, solid, rounded, microcystic image in the tail of the pancreas measuring 2.7 x 2.5 cm. The main pancreatic duct (MPD) was normal and no communication with the cysts was identified (Figures 2, 3).

**Figure 4:**
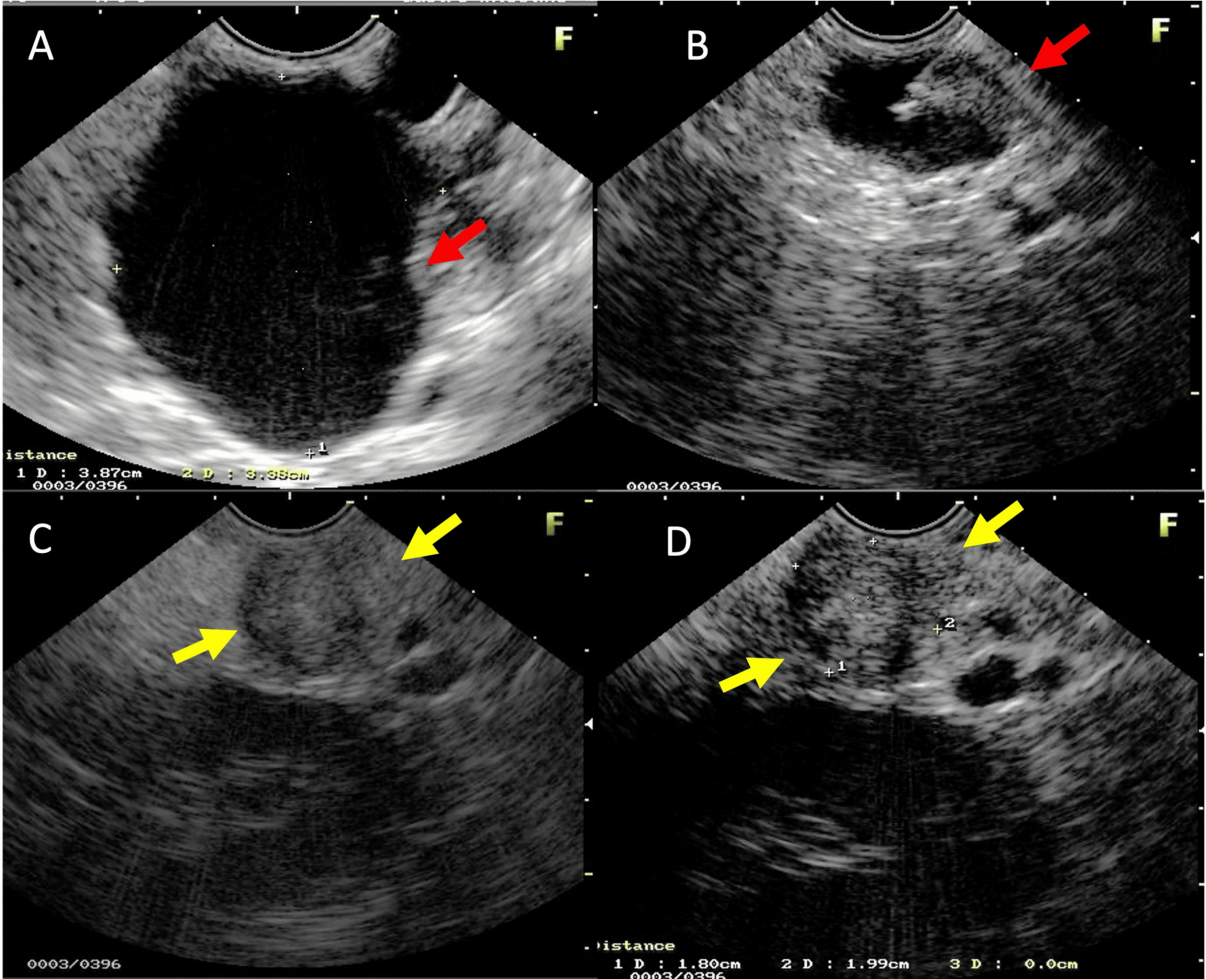
Endoscopic ultrasound-guided fine needle biopsy (EUS-FNB): (A) image of the cyst located in the transition between the body and tail of the pancreas (red arrow). (B) Image of the cyst with a decrease in volume during puncture with a 19G needle (red arrow). (C and D) Image of a hypoechoic, heterogeneous area with precise, rounded limits and microcystic areas inside measuring 1.9x1.8 cm.

The EUS confirmed the MRI/MRCP findings regarding the multiple pancreatic cysts, besides the 1.9 x 1.8 cm nodule located in the tail of the pancreas (Figure 4).

**Figure 5:**
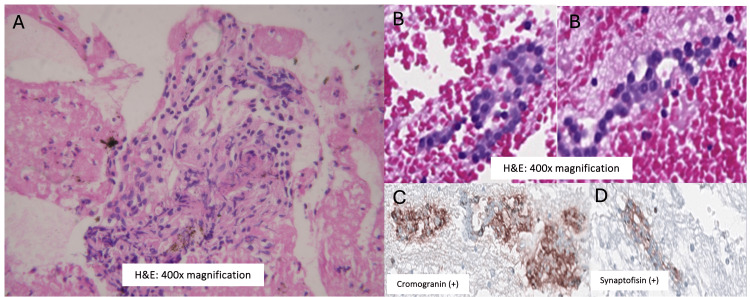
A: The microhistology confirmed of the cyst wall with macrophages, hemosiderin and saponified material, reinforcing the hypotheses of serous cyst neoplasia (SCN). B: The biopsy of the nodule revealed an infiltrative form of growth in a desmoplastic area, with mitotic activity, necrosis and no vascular invasion. Immunohistochemistry suspected pancreatic-neuroendocrine tumors (p-NET), with unidentified Ki 67, synaptophysin (+) and chromogranin (+ focal).

With a 19G needle, we punctured and emptied the 3.9 x 3.8 cm cyst located at the transition between the body and tail. A citrine yellow liquid was sent for biochemical study which revealed CEA 0.29 U/ml, CA 19-9 243 U/ml and amylase of 127U/ml. The results were suggestive of non-mucinous neoplasm (n-MN), confirmed by McH of the cyst wall with macrophages, hemosiderin and saponified material, reinforcing the hypotheses of SCN. The biopsy of the nodule revealed an infiltrative form of growth in a desmoplastic area, with mitotic activity, necrosis and no vascular invasion. The immunohistochemical examination suspected p-NET, with unidentified Ki 67, synaptophysin (+) and chromogranin (+ focal) (Figure 5).

This patient remained asymptomatic and was followed up with imaging exams for 24 months. After two years of follow-up, MRI/MRCP revealed growth of the tail nodule, rising from 1.9 to 2.9 cm, therefore, growth of 1.0 cm in two years, requiring total pancreatectomy. The pathological examination of the surgical specimen revealed SCN throughout the pancreatic gland, confirming the findings of the EUS-FNB performed two years before. The 3.0 cm nodule on the tail was compatible with MSNN with immunohistochemistry that showed Ki67 >10%, chromogranin (+ focal), synaptophysin (+) and CD56 (+). This patient remains in follow-up appointments for 12 years, with controlled diabetes mellitus.

Clinical case 2

**Figure 6:**
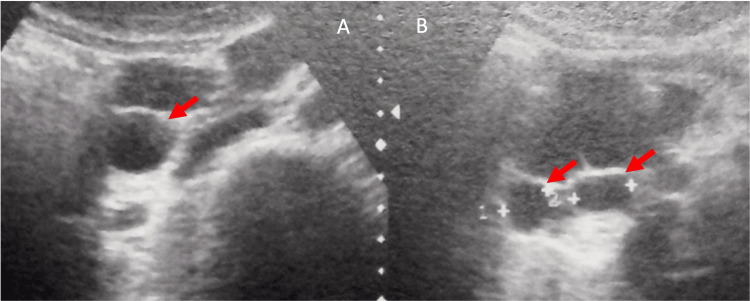
(A and B) Abdominal ultrasound: Several anechoic images, with rear acoustic reinforcement located in the head of the pancreas (red arrows).

A 41-year-old woman with abdominal pain accompanied by nausea and vomiting for 30 days. She reported three episodes of acute pancreatitis since the age of 12 years old, the first of which was severe and the others mild, but all of undefined etiology. The ultrasound at the time revealed small pancreatic cysts. At the age of 31, she underwent Fobi-Capella surgery as a treatment for morbid obesity. It was said that a first-degree relative had VHL. Laboratory tests for amylase, lipase, triglycerides, fasting blood glucose and glycated hemoglobin were normal. The abdominal ultrasound showed a normal gallbladder, and anechoic images scattered throughout the pancreatic parenchyma, with the largest measuring 5.2 x 3.2 cm in the longest axis and the smallest measuring between 1.5 and 1.8 cm (Figure 6).

**Figure 7:**
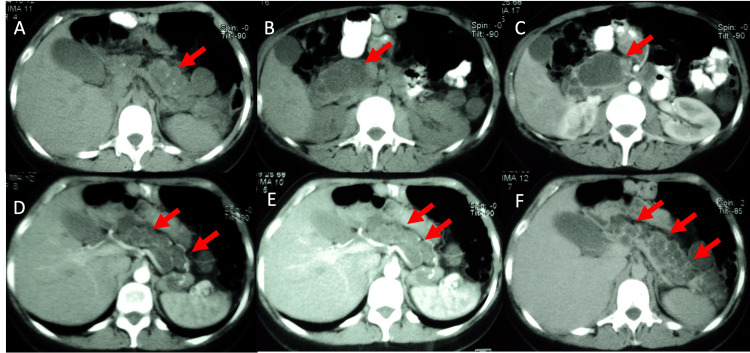
CT Imaging showing (A) numerous rounded hypodense images scattered throughout the pancreatic gland (red arrows). (B and C) hypodense images with well-defined walls, some of them loculated in the head of the pancreas (red arrows). (D, E and F) show different cut levels with hypodense areas of multiple sizes dispersed throughout the pancreatic parenchyma (red arrows).

The CT revealed preserved and shapeless pancreatic parenchyma with multiple cysts (Figure 7), the two largest being in the head (5.6 x 3.9 cm) and tail (4.7 x 4.6 cm).

**Figure 8:**
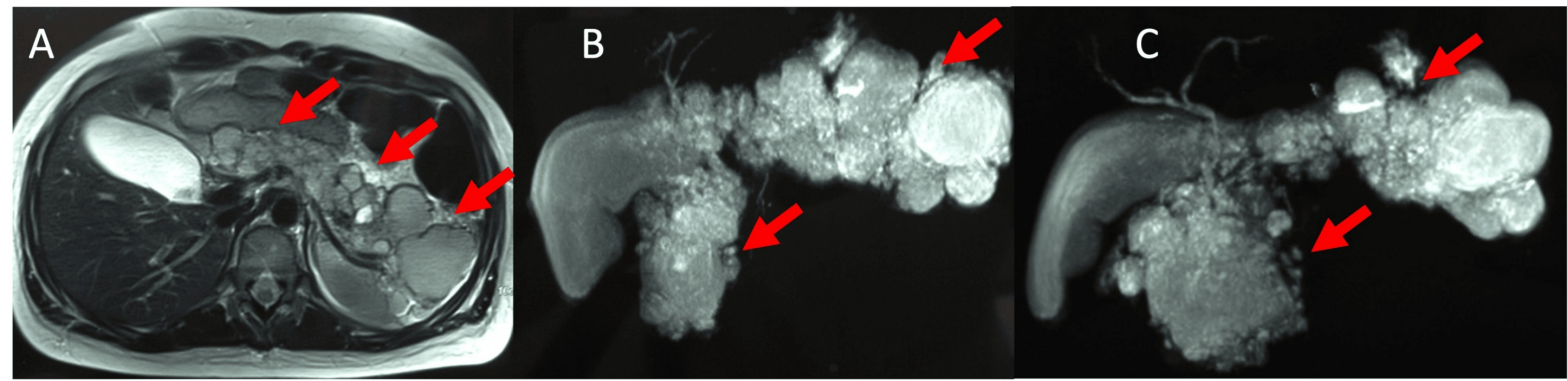
(A) MRI Imaging shows numerous cysts scattered throughout the pancreatic gland with no evidence of nodular images (red arrows). (B and C) Magnetic resonance cholangiopancreatography (MRCP) shows multiple cystic formations dispersed throughout the pancreatic gland with a large conglomerate of cysts located on the unciform process and tail of the pancreas (red arrows).

Patient underwent MRI/MRCP, which showed cystic formations with lobulated contours, thin septations, without vegetation. Also, two large cysts were identified, one on the head and the other on the tail of the pancreas. The MPD was visible only in a short segment of the body with normal caliber and without communication with the cystic formations (Figure 8).

**Figure 9:**
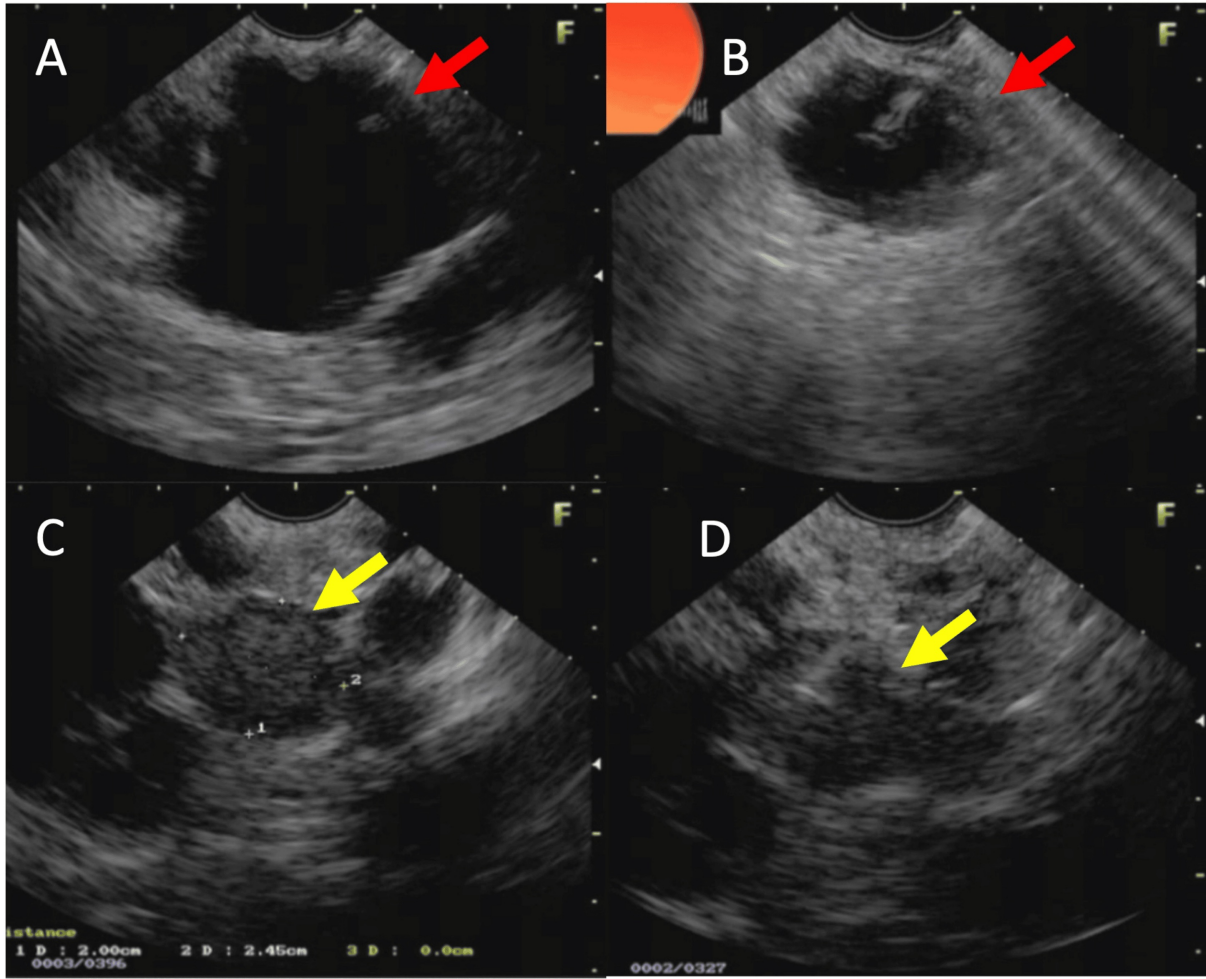
(A) Endoscopic ultrasound (EUS) imaging showing anechoic area with well-defined limits (red arrow). (B) Note the moment of EUS-fine needle biopsy (FNB) with a decrease in volume that can be identified in the image (A). (C) after complete emptying of the cyst (A) it was possible to identify a rounded, hypoechoic, heterogeneous image, with 2.4 x 2.0 cm and precise limits, containing microcystic areas inside (yellow arrow). (D) Moment of the EUS-FNB with 19G needle.

The EUS-FNB with a 19G needle was recommended to diagnose the type of cyst found in the image. After emptying the largest cyst in the body of the pancreas, EUS revealed a heterogeneous, hypoechoic nodule with microcystic areas measuring 2.3 × 2.1 cm, unidentified by CT and MRI, which was promptly submitted to EUS-FNB for tissue attainment (Figure 9).

**Figure 10:**
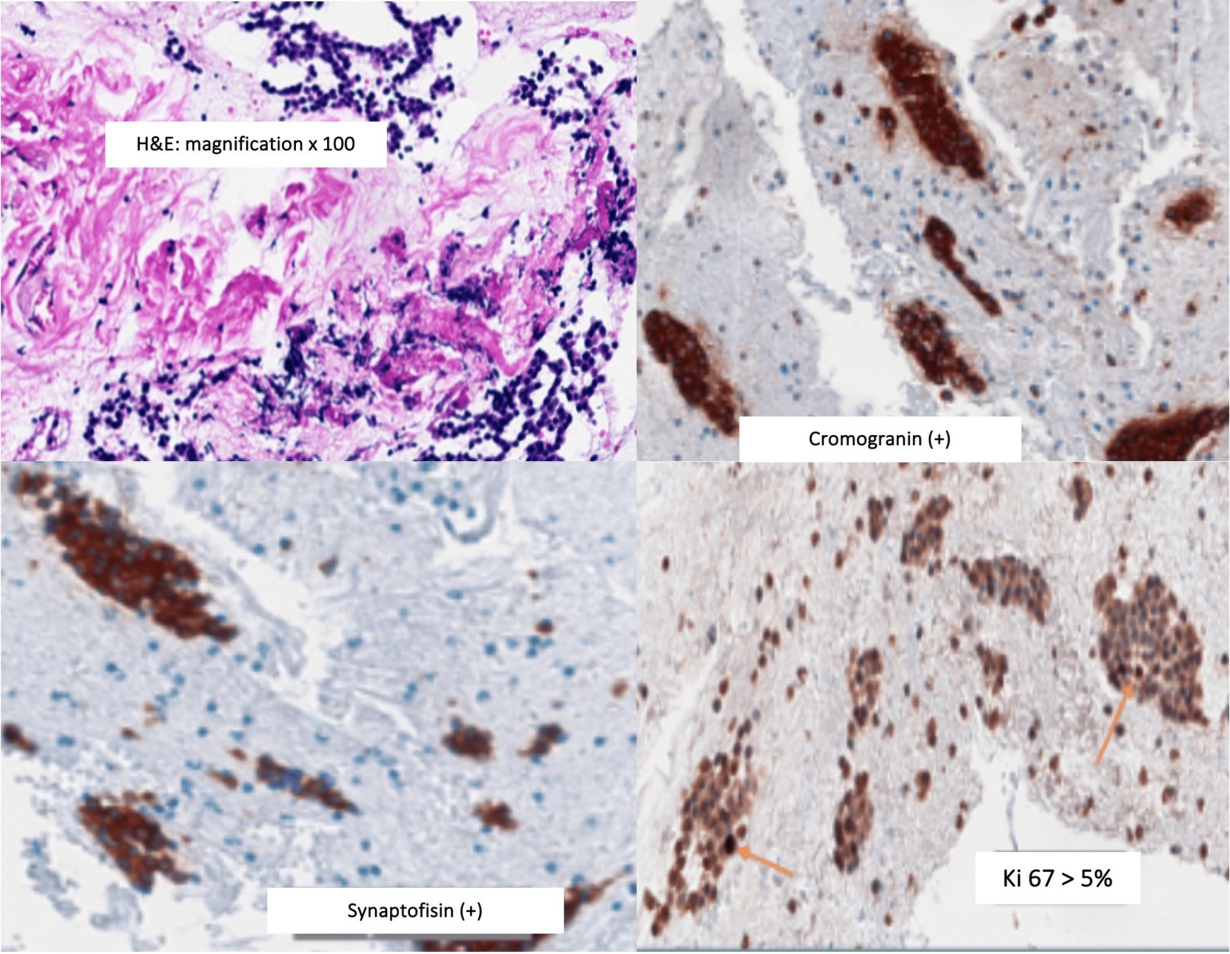
The pancreatic-neuroendocrine tumor (p-NET) with low histological grade, synaptophysin (+) and chromogranin (+) and Ki-67>5%.

The McH analysis associated with immunohistochemistry obtained by EUS-FNB confirmed the presence of a p-NET with low histological grade, synaptophysin (+) and chromogranin (+) and Ki-67>5% (Figure 10). The McH analysis of the cyst confirmed clusters of leukocytes and fibrin, with fluid analysis revealing CEA, CA19-9 and amylase levels of 3.6 ng/ml, 94.3 U/ml and 83U/L, respectively, which pointed to the diagnosis of an SCN. The patient attended appointments for five years, but was lost to follow-up due to a change of residence.

## Discussion

Von Hippel (1911) reported the first cases of retinal angiomatous lesions associated with cerebellar lesions that caused blindness in 1864. This disease was attributed to a hereditary condition discovered by Arvid Lindau (1926). Melmon and Rosen specified the eponym and diagnostic criteria of this disease [15]. Its incidence is one in 36,000 newborns [16]. The frequency of pancreatic involvement in VHL is common and ranges from 35% to 70% [16,17]. This condition is generally asymptomatic and does not require treatment. However, pancreatic cystic neoplasms with malignant potential must be excluded [18]. The two types of cystic neoplasms most frequently seen in VHL are simple cysts (70%) and SCNs (6-9%), while solid p-NETs are found in approximately 17%. The pancreatic adenocarcinoma is rare in VHL [1,19], as are endocrine or exocrine insufficiencies [20]. The majority of p-NETs in VHL are benign and non-functioning, with a malignancy rate ranging from 2% to 11%, different from the general population which is between 60% and 70% [18,21-23]. Although rare, due to the risk of malignancy, patients with VHL are candidates for periodic surveillance, according to clinical criteria [21], similar to the cases presented in this study.

Pancreatic involvement in VHL is usually detected between the ages of 23 and 56, similar to our patients. Generally, the growth of neoplasms identified in the pancreas is slow and the symptoms are infrequent. However, depending on the number, size and location of the lesions, abdominal pain and biliopancreatic obstruction may occur [1]. Typically, only one type of lesion (cystic or solid) is present. Another study found that out of the 122 patients, combined lesions were found in 11.5% and in 9% when one of the lesions was a p-NET [1]. Another author reported that only one out of 55 patients presented multiple cysts and p-NET combined [23]. Our patients presented synchronous lesions: SCN and MSNN in case 1 (mixed) and SCN and p-NET in case 2 (solitary), confirming previous reports.

The main strategic objective in VHL is to prevent metastasis. Only a few cases (8.2%) will require surgery as long as they are well monitored [1]. However, lesions with malignant potential, such as a mucinous neoplasm (MN) and/or an intraductal papillary mucinous neoplasia (IPMN), must be ruled out, despite being rare in this disease. Therefore, surgery is indicated in patients with these two types of lesions, with an indistinguishable or symptomatic cystic lesion [24]. On the other hand, solid lesions are treated according to their size and based on immunohistochemical characteristics. Patients with functioning p-NET (fp-NET) must undergo surgery, despite their size, due to the risk of malignancy and metastasis, although, in some cases, alternative treatments can be performed in these patients, such as radiofrequency [25]. In this article, we report the history of two patients with p-NET: one characterized as MSNN and the other with pure p-NET. The first asymptomatic patient presented typical findings of VHL associated with pancreatic and other organ lesions (eyes and brain). The second presented pancreatic cysts associated with episodes of acute pancreatitis. Both were followed for a long period of time with EUS being performed periodically.

Proper diagnosis of VHL-associated p-NETs must be made before deciding in favor of any treatment, which is often a challenge. The diagnostic accuracy and sensitivity of the diffusion-weighted (DW) MRI/MRCP are significantly higher than those of abdominal CT [14]. However, as performed in our patients, EUS-FNB should be the method of choice for the diagnosis and monitoring of solid pancreatic neoplasms identified in the course of VHL [24]. The decision of clinical follow-up was based on immunohistochemistry, indicating p-NET G1 in both cases. There are reports that the mortality rate due to metastasis of pNETs in VHL is around 0.3% [20], thus an indication for periodic surveillance [18,19,21]. Three criteria have been proposed to predict metastatic disease due to a p-NET in VHL: tumor size ≥ 3cm, exon 3 mutation, and growth capacity of the tumor (doubling time <500 days). If none of these criteria are present, follow-up can be performed with CT, MRI or EUS every two to three years, as seen in the cases from this study. If one of the criteria is present, imaging studies should be performed every six to 12 months. If two or more criteria are present, surgery is indicated [19]. Based on these criteria, we decided to follow up in both cases. Between MRI/MRCP, CT and EUS, EUS is preferable for solid lesions, allowing a better distinction between SCN and p-NET [12,26]. The analysis of the cystic fluid obtained by EUS-FNB has an accuracy of approximately 93% to distinguish an MN from an n-MN, but the best results for the diagnosis of an SCN or a p-NET come from EUS-FNB with microhistology [12]. Among the patients in this study, amylase, CEA and CA19-9 were normal, drawing attention to n-MN.

The SCNs are cystic neoplasms that frequently occur in patients with VHL. Occasionally, other tumors can be found simultaneously with SCNs in surgical specimens, such as p-NET, pancreatic adenocarcinoma, IPMN, and metastatic tumors [27]. p-NETs are the most common solid tumors and, when found with SCN, the mixed tumor is called MSNN. In a cohort of 193 SCNs, the simultaneous occurrence of SCN and p-NET appeared in 6% (12/193) of all cases [27]. Including the first report in 1991 [28], 32 similar cases of MSNN have been published in PubMed English literature until June 2019. Based on the distribution pattern of SCNs and p-NET, MSNN has been classified into four subtypes: (1) Diffuse: the entire pancreas is occupied by SCNs associated with one or more pNETs, located in any region of the pancreas; (2) Mixed: the two different tumors combine together into a single mass and cannot be divided distinctly; (3) Solitary: the two different components coincide in the pancreas without any mixing; and (4) Coalition: the two types of neoplasms separate from each other in most of the pancreas, but with a partially mixed or overlapping area [2,11].

Reid et al. conducted an analysis that showed that among the MSNNs, 41% (9/22) were the diffuse type, 27% (6/22) were the mixed type, 23% (5/22) were the solitary type, and 9% (2/22) were the coalition type. Most SCNs can be easily differentiated from p-NETs by histology without the need for ancillary and immunohistochemical examinations. However, in rare cases, SCN consists of small uniform nests or tubules with minimal lumen formation, specifically the solid type of SCN or solid serous adenoma [27]. Malignant behavior is extremely rare in SCN [28]. In fact, p-NETs are considered low-grade malignancy; therefore, the prognosis of MSNN depends on the p-NET component. These cases may be misdiagnosed as p-NET [29]. Thus, differential diagnosis between an SCN and a p-NET can be challenging, especially in small biopsies such as EUS-FNB, as long as the choice is by cytology. However, SCN can be distinguished from p-NET based on the lack of reactivity of markers for neuroendocrine cells (chromogranin A) [30]. The recent 2017 WHO classification divided p-NETS into well-differentiated and undifferentiated, based on evidence from genetic data [31]. Also, p-NETs are classified on a scale of 1-3 based on mitotic rates and Ki67 proliferation index as follows: p-NET G1 with <2 mitoses/10 HPF and <3% Ki67 index; p-NET G2 with 2-20 mitoses/10 HPF and Ki67 index of 3%-20%; and p-NET G3 with >20 mitoses/10 HPF and Ki67 index >20%. Thus, in the pathological images of our MSNN cases, the p-NET component of the first case can be classified as a G2 and the second one as a G1. Lymph node metastases can occur even when the diameter of the p-NET is less than 0.5 cm [32]. Therefore, the pathologist must perform a careful macroscopic examination of the specimen to exclude the presence of a concomitant p-NET in samples from an SCN.

There are two hypotheses that explain the formation of MSNN: common progenitor cells and distinct progenitor cells. For the first hypothesis, both SCN and p-NET would derive from a common pluripotent stem cell [33], supported by the observation of biphasic differentiation in a pancreatic lesion, such as nesidioblastosis, a non-neoplastic disease with epithelial and endocrine origin with coincident differentiation [34]. The identification of neurosecretory granules, glycogen and intermediate filaments within the same tumor cells by ultrastructural examination would explain cases in which both tumors are intimately mixed, as in the mass found in case 1, but cannot explain those with separate lesions, as in the second case presented [35]. For the other hypothesis, MSNN would derive from two different cell types, arising from a predisposing genetic disorder, such as VHL [36]. This idea can be supported by the occurrence of genetic mutations in SCNs being different from those in p-NET [37], indicating a distinct pathogenesis of the tumors. In summary, further studies, including molecular analyses, are still needed to elucidate the pathogenesis and origin of MSNN. Importantly, if an MSNN is confirmed in a patient without suspected VHL, other parts of the body need to be evaluated, and genetic testing performed to exclude VHL.

## Conclusions

The authors report two unprecedented cases of MSNN in which both patients were diagnosed by EUS-FNB with microhistology: one belonging to the mixed type and the other, solitary, with SCN involving the entire pancreas. Due to its relationship with VHL, which generally presents benign lesions and does not require surgical treatment, both were initially subjected to surveillance with MRI and EUS. This monitoring strategy was only possible due to the diagnosis obtained by EUS-FNB associated with McH. Surgical treatment was appropriately indicated due to the increase in volume of the mixed MSNN during follow-up with imaging exams, while in the other case of MSNN the proposed option was “Watch and Wait” after adequate diagnosis.
